# A Rare Case of Choriocarcinoma With Myometrial Invasion Mimicking Invasive Mole: Case Report

**DOI:** 10.1002/ccr3.71094

**Published:** 2025-10-08

**Authors:** Shaghayegh Vandadi, Afsaneh Tehranian, Nasim Zarifi, Akram Seifollahi, Amirhossein Hajialigol

**Affiliations:** ^1^ Department of Obstetrics and Gynecology, Arash Women's Hospital, School of Medicine Tehran University of Medical Sciences Tehran Iran; ^2^ Alborz Office of Universal Scientific Education and Research Network (USERN), Alborz University of Medical Sciences Karaj Iran

**Keywords:** Beta‐hCG, choriocarcinoma, fertility preservation, gestational trophoblastic neoplasia, invasive mole, myometrial invasion

## Abstract

Gestational trophoblastic neoplasia (GTN) comprises a group of rare tumors arising from placental trophoblastic tissue, including choriocarcinoma and invasive mole. We present a rare case of choriocarcinoma with isolated myometrial invasion in a 30‐year‐old woman with a history of molar pregnancy and term delivery. The clinical presentation mimicked an invasive mole, with elevated β‐hCG levels and imaging revealing a vascular myometrial mass. Following a failed dilation and evacuation, and in light of the patient's desire to preserve fertility, conservative surgery via laparotomy was performed. Pathology confirmed choriocarcinoma with myometrial and vascular invasion. Postoperative treatment with actinomycin‐D chemotherapy led to remission, and the patient remained disease‐free at two‐year follow‐up. This case emphasizes the importance of histopathological confirmation in GTN diagnosis and illustrates that conservative surgical management, when appropriate, can achieve both oncologic control and fertility preservation.

**Summary**

- This rare case of choriocarcinoma with isolated myometrial invasion mimicking invasive mole highlights the importance of histopathological confirmation for accurate diagnosis.
- Conservative surgery combined with chemotherapy can be an effective fertility‐preserving approach in selected cases of GTN, provided careful staging, individualized planning, and close long‐term follow‐up are maintained.

## 1 Introduction

Gestational trophoblastic neoplasia (GTN) encompasses a spectrum of rare, potentially malignant disorders originating from the placental trophoblast. These conditions can arise after any type of gestation, though they are most commonly associated with molar pregnancies [1, 2, 3]. GTN is characterized by abnormal trophoblastic proliferation and continued production of human chorionic gonadotropin (hCG), which serves as a key diagnostic and follow‐up biomarker [4, 5].

The four primary histological subtypes of GTN include: invasive mole, choriocarcinoma, placental site trophoblastic tumor (PSTT), and epithelioid trophoblastic tumor (ETT). While these types share a common trophoblastic origin, they differ significantly in clinical behavior, metastatic potential, and hCG production levels [6]. Invasive mole is typically a sequela of complete hydatidiform mole and tends to remain confined to the uterus. In contrast, choriocarcinoma is highly malignant and often metastatic, whereas PSTT and ETT may show lower hCG levels and are relatively chemo‐resistant [7].

The incidence of GTN varies globally due to differences in diagnostic practices and ethnic predispositions. For example, the rate is estimated to be 0.4–1 per 1000 pregnancies in North America and Europe, but may reach 12.5 per 1000 pregnancies in parts of Asia [8]. In Iran, GTD affects approximately 5.4 per 1000 deliveries, with 6.4% of complete moles advancing to invasive mole and 2.7% to choriocarcinoma [9]. Established risk factors include a prior molar pregnancy, maternal age over 40, and Asian ancestry [10, 11]. The diagnosis of GTN relies on clinical presentation, serial quantitative β‐hCG (mIU/mL) measurements, and pelvic imaging. Patients often present with abnormal uterine bleeding or persistently elevated hCG levels following pregnancy [11]. Ultrasonography may reveal intrauterine masses with increased vascularity, and further imaging may be warranted based on metastasis risk. Following confirmation, patients are staged using the FIGO system and scored using the WHO prognostic scoring system to determine the risk category and appropriate treatment protocol [12].

Prompt recognition and accurate classification of GTN are essential for optimal management and fertility preservation. Most cases respond well to chemotherapy, especially when diagnosed early. The present case highlights an unusual clinical scenario in which a choriocarcinoma presented with imaging findings suggestive of an invasive mole, leading to surgical exploration and fertility‐sparing treatment. This underscores the importance of histopathological confirmation and individualized therapeutic planning in suspected GTN.

## 2 Case History and Clinical Examination

**FIGURE 1:**
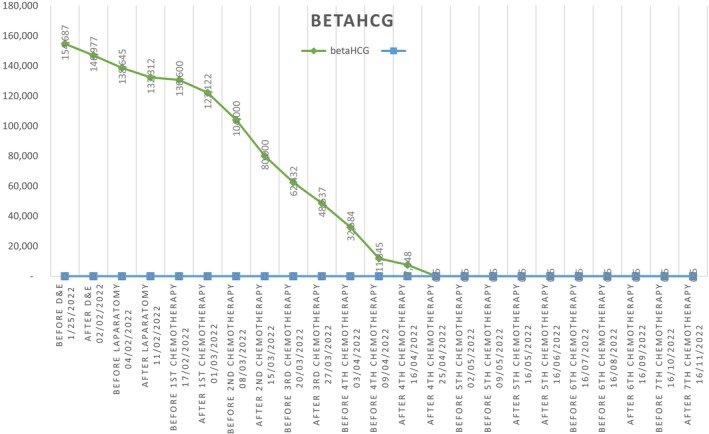
Beta‐HCG chart before and after D&E and chemotherpy.

**FIGURE 2:**
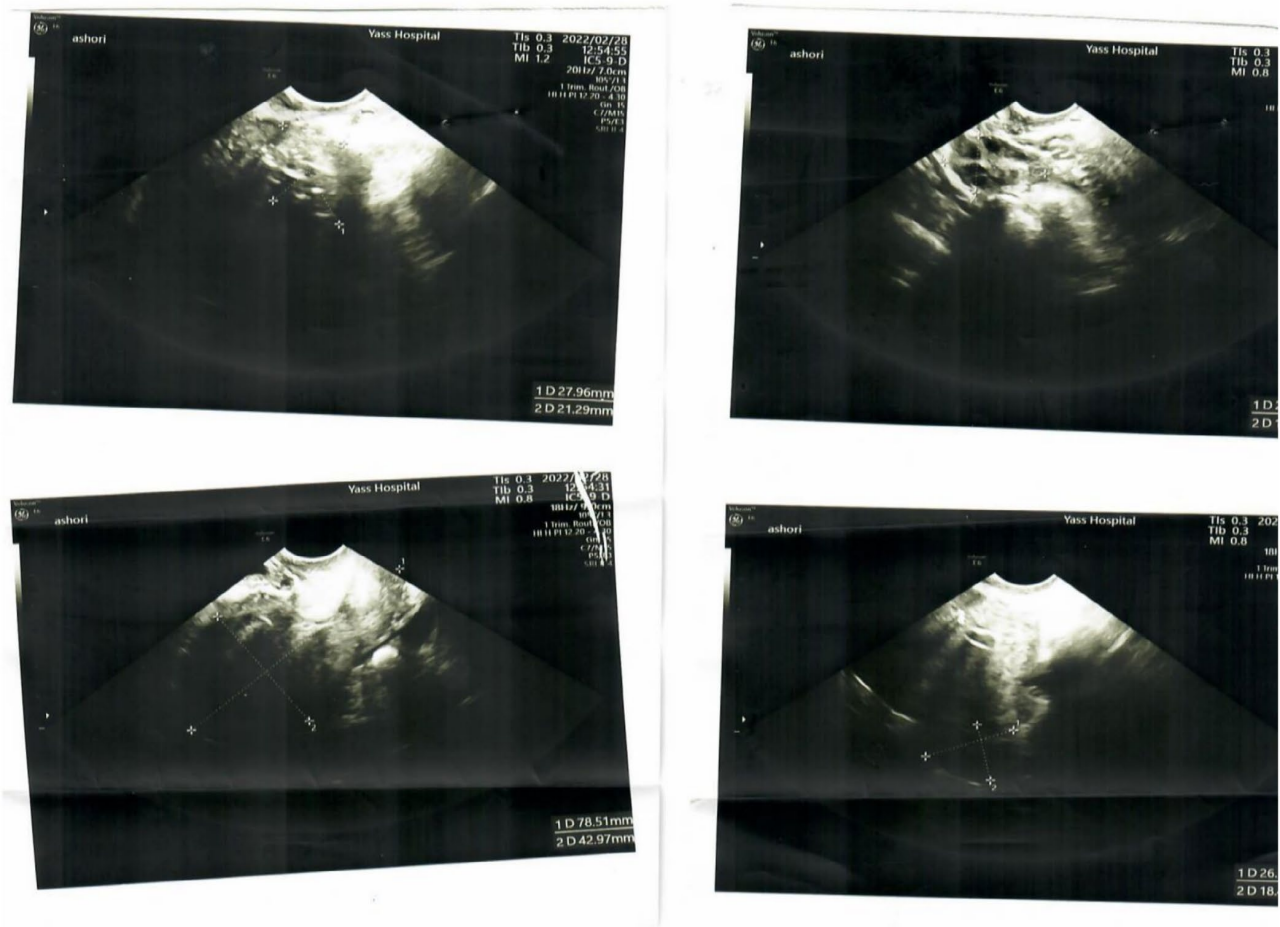
Subsequent transvaginal ultrasound revealed a heterogeneous echogenic mass (29 × 26 × 22 mm) with irregular cystic regions in the anterior portion of the uterus at the inferior part of the cornea that has high flow and low resistance vascular Doppler within the lesion. Myometrial thickness at the site of the lesion was 2 mm.

A 30‐year‐old woman, 1011 presented at Arash woman's hospital due to vaginal bleeding from 2 weeks ago and positive Quantitative plasma Beta‐hCG. Her first pregnancy, a molar pregnancy, occurred 4 years ago, which was managed with Dilation and evacuation (D&E). Her second pregnancy, 3 years ago, resulted in a term delivery via cesarean section without any complications. The patient had no other previous medical or surgical history, and she didn't use any contraception methods. Vaginal examination revealed no discoloration, swelling, or discharge. The cervix appeared healthy and firm with minimal bleeding upon speculum examination. She didn't have any abdominal pain. Her initial β‐hCG (mIU/mL) level was 150,000. Complete Blood Count, liver enzymes, thyroid, kidney, and other tests were normal. Trans‐vaginal ultrasound showed increased endometrial thickness with no significant lesion. Dilation and evacuation (D&E) surgery was performed, and an endometrial tissue sample was sent for pathology examination (Figure 1). One week later, the β‐hCG plasma level was 146,977, and the pathology report indicated only decidualized endometrial tissue without any trophoblastic component. Subsequent transvaginal ultrasound revealed a heterogeneous echogenic mass (29 × 26 × 22 mm) with irregular cystic regions in the anterior portion of the uterus at the inferior part of the cornua that has high flow and low resistance vascular doppler within the lesion. Myometrial thickness at the site of the lesion was 2 mm (Figure 2).

Metastatic workup revealed no metastasis in other organs. Based on the findings of trans‐vaginal ultrasound and elevated β‐hCG plasma level, the diagnosis of GTN was made. According to the anatomical staging system and prognostic scoring system developed by the FIGO, the patient was diagnosed with stage I (restricted to uterus) GTN with a total score of 7 (2 points for pre‐treatment serum β‐hCG, 1 point for antecedent term pregnancy, and 4 points from interval > 12 months). After consultation with other physicians and the patient, it was decided not to proceed with total abdominal hysterectomy surgery due to the patient's desire to conceive again. Instead, a laparotomy was performed. Following a Pfannenstiel incision, a hysterotomy incision was made on the uterus. The molar trophoblastic tissue was observed in the myometrium with invasion into the endometrium.

**FIGURE 3:**
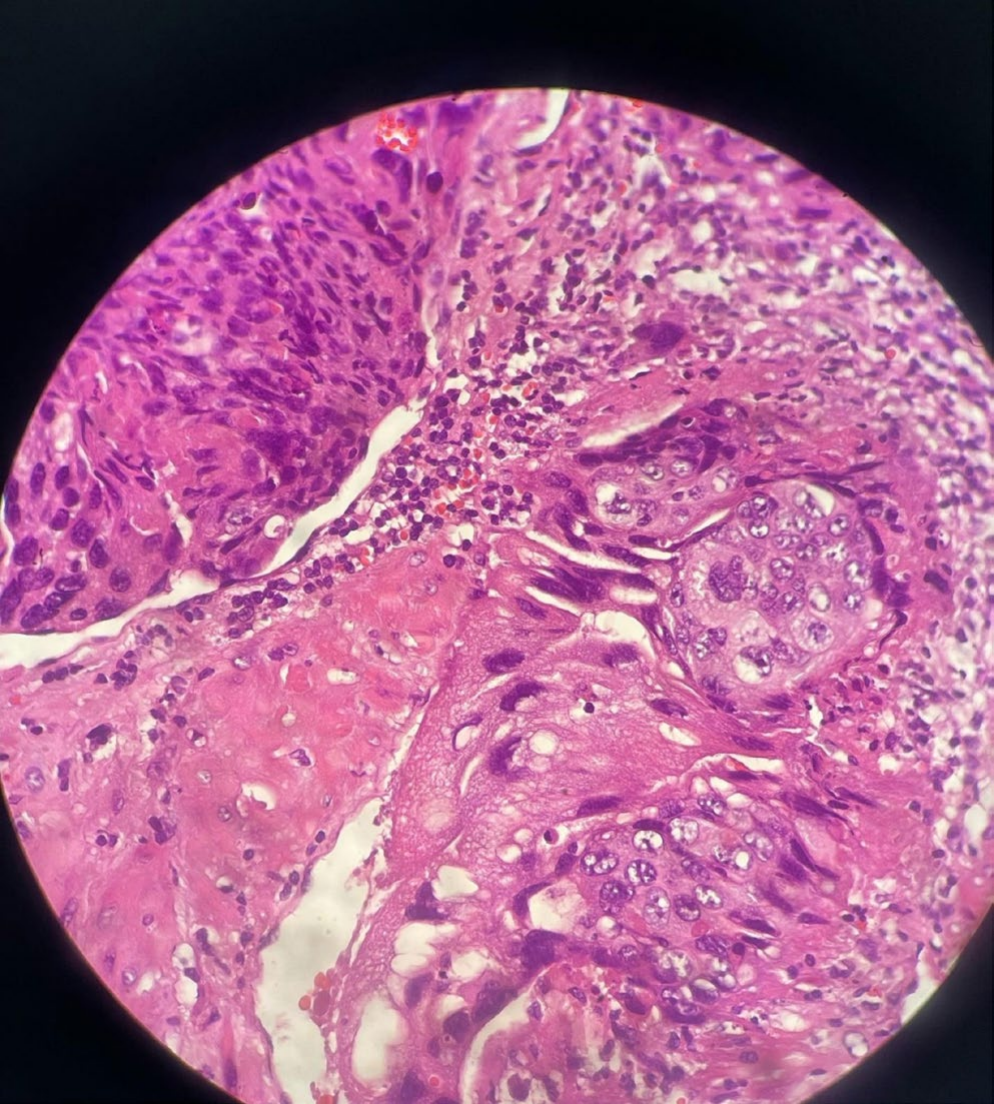
The pathology reported choriocarcinoma with myometrial and vascular invasion.

The myometrial mass was completely excised, and the remaining myometrial tissue was debrided and cleaned with normal saline, and the sample was sent for pathology. The pathology reported choriocarcinoma with myometrial and vascular invasion. (Figure 3) The patient received 7 doses of chemotherapy with actinomycin‐D. Serial β‐hCG plasma level was monitored weekly until it became undetectable. The patient also underwent serial examinations and two methods of contraception (OCP and condom). After 2 years of follow‐up, no recurrence was observed.

## 3 Differential Diagnosis, Investigations and Treatment

Upon initial presentation with prolonged vaginal bleeding and markedly elevated β‐hCG levels, the most likely diagnosis was gestational trophoblastic neoplasia (GTN) [13]. Given the patient's prior history of molar pregnancy and the presence of an intrauterine mass on ultrasound, an invasive mole was initially considered. However, other differential diagnoses such as choriocarcinoma, retained products of conception, or even less common uterine pathologies were also taken into account. Further investigation was critical to narrowing the diagnosis. A transvaginal ultrasound revealed a heterogeneous echogenic mass in the anterior myometrium with high‐flow, low‐resistance Doppler signals—findings more characteristic of a neoplastic process with vascular invasion. Despite undergoing dilation and evacuation (D&E), the pathology report only showed decidualized endometrial tissue, with no evidence of trophoblastic cells, raising concern for an extra‐endometrial lesion. A second ultrasound confirmed persistent abnormal findings and thin myometrial tissue at the site of the lesion. To assess the extent of disease, a full metastatic workup was conducted, including thoracic and abdominal imaging, which showed no evidence of spread. Based on clinical presentation, β‐hCG levels, and the anatomical findings, the patient was diagnosed with stage I GTN confined to the uterus. The FIGO prognostic scoring system assigned a total score of 7, placing her in the high‐risk category. In light of her strong desire to preserve fertility, a multidisciplinary decision was made to avoid hysterectomy. Instead, the patient underwent a fertility‐preserving laparotomy. Through a Pfannenstiel incision, a localized hysterotomy was performed, and the myometrial lesion was identified and excised with care to preserve surrounding uterine tissue. Intraoperatively, no endometrial mass was noted, but the lesion appeared infiltrative, raising suspicion for choriocarcinoma.

Postoperative pathology confirmed the diagnosis of choriocarcinoma with both myometrial and vascular invasion. Following surgery, the patient was treated with single‐agent chemotherapy using actinomycin‐D. Treatment was initiated promptly and monitored closely with serial β‐hCG measurements to assess response.

## 4 Conclusion and Results (Outcome and Follow‐Up)

This case underscores the importance of considering choriocarcinoma in the differential diagnosis when confronted with persistent elevation of β‐hCG and an intrauterine lesion unresponsive to standard D&E. Although initially suspected to be an invasive mole, histopathological confirmation following conservative surgery revealed choriocarcinoma with myometrial and vascular invasion. The patient completed seven cycles of actinomycin‐D chemotherapy, with weekly β‐hCG monitoring. Notably, β‐hCG levels began to decline significantly after the third dose and became undetectable by the seventh cycle. Four consecutive negative β‐hCG results confirmed biochemical remission. Post‐treatment follow‐up involved both clinical examinations and continued β‐hCG surveillance. The patient adhered to dual contraceptive methods (oral contraceptives and condoms) during the surveillance period. At the two‐year mark, she remains recurrence‐free and in good general health, with preserved reproductive potential. This case highlights the value of individualized treatment approaches in GTN, especially for women seeking fertility preservation. Conservative surgery followed by targeted chemotherapy can lead to successful outcomes in select high‐risk cases when guided by careful staging, accurate diagnosis, and close follow‐up.

## 5 Discussion

This case illustrates a rare presentation of choriocarcinoma with myometrial and vascular invasion, initially suspected to be an invasive mole based on ultrasound findings. Accurate classification of GTN is critical and begins with staging using the FIGO criteria and the WHO prognostic scoring system. These tools stratify patients based on metastatic spread and key prognostic indicators, such as hCG levels and time since antecedent pregnancy, to guide optimal treatment approaches [14, 15].

A particularly challenging diagnostic entity in GTN is the complete hydatidiform mole coexistent with a normal fetus (CHMCF), which carries a 16%–50% risk of progression to GTN. This rare form of twin pregnancy—occurring in 1 in 20,000 to 1 in 100,000 pregnancies—presents unique obstetrical risks and diagnostic complexities. Because β‐hCG is less specific during viable pregnancies, early identification via distinct ultrasonographic markers is essential for timely diagnosis and intervention [16, 17].

A particularly important diagnostic challenge in gestational trophoblastic disease is the rare occurrence of a complete hydatidiform mole coexisting with a normal fetus (CHMCF). This entity poses a significant clinical concern, as it is associated with a 20%–50% risk of progression to GTN [18]. Early detection is complicated by nonspecific ultrasound findings in the first trimester, which may resemble more benign conditions such as subchorionic hematoma. Compounding the difficulty, β‐hCG levels—typically a key diagnostic marker—are less reliable during ongoing pregnancies due to physiological elevation, thus limiting their diagnostic utility in these cases [19]. For this reason, detailed ultrasonographic assessment becomes essential for timely recognition. It is also crucial to distinguish CHMCF from other conditions with overlapping imaging features, such as partial hydatidiform mole (PHM), placental mesenchymal dysplasia (PMD), and placental chorioangioma, as each has distinct prognostic and therapeutic implications. Accurate differentiation guides appropriate management strategies and optimizes outcomes for both the patient and the fetus. The WHO Prognostic Scoring System categorizes patients as low‐ or high‐risk based on clinical and pathological parameters. Patients with a score ≥ 7 are considered high risk and are more likely to require multiagent chemotherapy. Scores ≥ 12 signify ultra‐high‐risk status, often associated with poor outcomes. For example, Kong et al. reported a five‐year survival of only 68% in these ultra‐high‐risk cases [20].

Modern management of GTN, particularly with the use of actinomycin‐D and methotrexate, has drastically improved prognosis. Most cases can now be treated successfully while preserving fertility, especially when diagnosed early. While surgery, including dilation and curettage or hysterectomy, was historically primary, chemotherapy now represents the mainstay of care except for chemoresistant subtypes like PSTT and ETT [21, 22]. Surgical intervention remains important in select cases, particularly for those with uncontrolled bleeding or no desire for future fertility [23].

In this patient, despite initial misclassification, fertility‐preserving laparotomy successfully removed the lesion. Postoperative histopathology confirmed choriocarcinoma, and the patient responded well to seven cycles of actinomycin‐D. Long‐term monitoring confirmed remission with no recurrence after 2 years. While laparotomy is not standard for GTN, this case underscores its potential value in selected cases. Future studies should evaluate the reproductive outcomes and complications such as uterine adhesions or placental abnormalities following this surgical approach.

In summary, in our specific case, there was no endometrial mass due to normal suction curettage of the endometrium, and there was no reduction in β‐hCG titration after the procedure. Hence, because of the patient's reluctance for hysterectomy and the couple's desire to have more children, we proceeded with myometrial laparotomy and the resection of the 3 cm myometrial mass, followed by debridement and cleaning the area with sodium chloride. Due to the pathology report emphasizing choriocarcinoma with vascular and myometrial invasion, the patient received seven doses of monochemotherapy with actinomycin‐D. Chemotherapy continued until four consecutive negative β‐hCG titrations were detected. There were negative β‐hCG titrations after three doses of chemotherapy. The patient received two forms of contraception (condom and contraception pills) for 2 years, and there was no recurrence after 2 years of follow‐up.

## Author Contributions

**Shaghayegh Vandadi:** conceptualization, supervision, writing – original draft, writing – review and editing. **Afsaneh Tehranian:** conceptualization, supervision, writing – original draft, writing – review and editing. **Nasim Zarifi:** conceptualization, supervision, writing – original draft. **Akram Seifollahi:** writing – review and editing. **Amirhossein Hajialigol:** writing – review and editing.

## Consent

Written informed consent was obtained from the patient for publication of this case report and accompanying images. A copy of the written consent is available for review by the Editor‐in‐Chief of this journal on request.

## Conflicts of Interest

The authors declare no conflicts of interest.

## Data Availability

The data that support the findings of this study are available from the corresponding author, [Afsaneh Tehranian], upon reasonable request.
